# Grading system utilising the total score of Oxford classification for predicting renal prognosis in IgA nephropathy

**DOI:** 10.1038/s41598-021-82967-x

**Published:** 2021-02-11

**Authors:** Yoei Miyabe, Kazunori Karasawa, Kenichi Akiyama, Shota Ogura, Tomo Takabe, Naoko Sugiura, Momoko Seki, Yuko Iwabuchi, Norio Hanafusa, Keiko Uchida, Kosaku Nitta, Takahito Moriyama

**Affiliations:** 1 Department of Nephrology, Tokyo Women’s Medical University, 8-1 Kawada-Cho, Shinjuku-ku, Tokyo, 1628666 Japan; 2 Department of Blood Purification, Tokyo Women’s Medical University, 8-1 Kawada-Cho, Shinjuku-ku, Tokyo, 1628666 Japan

**Keywords:** Nephrology, Risk factors

## Abstract

The Oxford classification of IgA nephropathy (IgAN) can evaluate each MEST-C score individually. We analysed a new grading system that utilised the total MEST-C score in predicting renal prognosis. Altogether, 871 IgAN patients were classified into three groups using the new Oxford classification system (O-grade) that utilised the total MEST-C score (O-grade I: 0–1, II: 2–4, and III: 5–7 points), and the 10-year renal prognosis was analysed. The clinical findings became significantly severer with increasing O-grades, and the renal survival rate by the Kaplan–Meier method was 94.1%, 86.9%, and 74.1% for O-grades I, II, and III, respectively. The hazard ratios (HRs) for O-grades II and III with reference to O-grade I were 2.8 (95% confidence interval [CI] 1.3–6.0) and 6.3 (95% CI 2.7–14.5), respectively. In the multivariate analysis, mean arterial pressure and eGFR, proteinuria at the time of biopsy, treatment of corticosteroids/immunosuppressors, and O-grade (HR 1.63; 95% CI 1.11–2.38) were the independent factors predicting renal prognosis. Among the nine groups classified using the O-grade and Japanese clinical-grade, the renal prognosis had an HR of 15.2 (95% CI 3.5–67) in the severest group. The O-grade classified by the total score of the Oxford classification was associated with renal prognosis.

## Introduction

IgA nephropathy (IgAN) has been recognised as the most common chronic glomerulonephritis with a mild to poor prognosis depending on the clinical and pathological backgrounds^1,2^. To determine the appropriate treatments to prevent progression, it is essential to predict renal prognoses more accurately according to the clinical and pathological findings^3,4^. Therefore, many classifications have been developed to predict the renal prognosis of patients with IgAN^5^. Recently, in Japan, two major classifications of IgAN, the Japanese histological classification^6,7^ and the Oxford classification^5,8,9^, which was the first international pathological classification, have been frequently used. In the Japanese histological classification, multivariate logistic regression analysis indicated that the independent risk factors related to the renal prognosis were cellular and fibrocellular crescents indicating acute glomerular lesions, global sclerosis, and segmental glomerulosclerosis, and fibrous crescents indicating chronic glomerular lesions. Then, the ratio of these glomerular lesions to the total number of glomeruli was classified into four grades, with every 25% as a lumped grading system^6^. Moreover, this histological classification combined with the clinical classification (C-grade) according to the estimated glomerular filtration rate (eGFR) and proteinuria was referred to as the Japanese histological classification to predict the prognosis of IgAN^7,10^. On the other hand, in the Oxford classification, the pathological findings of mesangial hypercellularity (M), endocapillary hypercellularity (E), segmental glomerulosclerosis (S), and tubular atrophy/interstitial fibrosis (T) were selected through univariate and multiple regression analyses, and the Cox proportional hazard regression model and cellular/fibrocellular crescent formation (C) were additionally selected in 2016^5,8,9^. In this classification, each pathological finding can be assessed individually as a split system, and there have been many validation studies on this classification that have assessed its efficacy in evaluating the prognosis of IgAN^11–16^.

The strong point of the lumped grading system, such as the Japanese histological classification, is that it evaluated the total disease activities and predicted the prognosis by using these data; however, assessing the details of each pathological finding is difficult when using this grading system. However, in the split system, such as the Oxford classification, evaluating the details of each pathological finding is easy, but it is challenging to evaluate the total disease activity and predict the precise prognosis of patients with IgAN. To the best of our knowledge, no clinical research has evaluated the MEST-C score comprehensively, similar to the lumped grading system, or has investigated the usefulness of the combination of the Oxford classification and the lumped grading system. Therefore, we hypothesised that, if the total score of each MEST-C score can be used for predicting more precise renal prognosis, it might be possible to resolve the limitation of the Oxford classification. Furthermore, the Oxford classification only evaluated the pathological findings, and it might be more useful if it could predict the renal prognosis as well, by combining this grading system with the Japanese histological classification that utilised clinical findings. In this study, we aimed to introduce our new grading system (namely O-grade) that utilised the total MEST-C score in the Oxford classification and to analyse whether the O-grade was related to renal prognosis. We also investigated whether the combination of the O-grade and clinical grade of the Japanese classification was related to the renal prognosis. Based on these analyses, we aimed to determine further whether the Oxford classification can more straightforwardly predict the renal prognosis of patients with IgAN.

## Results

### Baseline characteristics

The patients’ baseline characteristics are shown in Table 1a. The patients’ median age at the time of renal biopsy was 31 years, and 40% of the patients were men. The median proteinuria level was 0.68 g/day, and the median eGFR was 77.0 ml/min/1.73 m^2^. For the Oxford classification, the rates of each variable were 49% for M1, 45% for E1, 72% for S1, 22% and 6% for T1 and T2, respectively, and 42% and 5% for C1 and C2, respectively. The mean total score of the Oxford classification was 2.5 points. At a median follow-up period of 8 years after the renal biopsy, 115 patients (13%) progressed to end stage renal disease (ESRD).

**Table 1:** Clinical and laboratory characteristics of the patients diagnosed with IgA nephropathy at the time of renal biopsy, and the patients classified by O-grades.

Characteristic variable	a	b			*P *value
	All patients	O-grade I	O-grade II	O-grade III	
Number of patients, n [male, n (%)]	871 [356 (41)]	260 [101 (38.9)]	525 [220 (41.9)]	86 [35 (40.7)]	0.7131
**At the time of biopsy**					
Age (year)	31 (24–41)	30 (24–42)	30 (24–40)	34.5 (27–46.3)	0.006
Duration from onset to biopsy (year)	3 (1–8)	3 (1–8)	4 (1–8)	3 (1–8)	0.73
BMI (kg/m^2^)	21.3 (19.6–23.5)	21.4 (19.4–23.6)	21.2 (19.6–23.4)	21.7 (19.8–24.1)	0.38
MAP (mm Hg)	89.7 (81.3–99)	88 (80.2–97)	89.3 (80.7–99.3)	95 (86.6–106)	< 0.001
Proteinuria (g/day)	0.68 (0.3–1.4)	0.34 (0.14–0.69)	0.78 (0.4–1.49)	1.73 (0.9–3.1)	< 0.001
Haematuria (/HPF)	20 (10–50)	20 (10–50)	30 (10–50)	30 (10–100)	0.015
Serum creatinine (mg/dL)	0.79 (0.67–1.0)	0.78 (0.64–0.98)	0.78 (0.68–1.0)	0.96 (0.77–1.29)	< 0.001
eGFR (mL/min/1.73 m^2^)	77.0 (60.0–95.6)	79.6 (65.5–96.9)	78.0 (60.4–98.0)	59.0 (48.8–75.7)	< 0.001
BUN (mg/dL)	14.8 (12.2–17.9)	14.4 (11.9–16.8)	14.7 (12.1–17.8)	17.6 (13.6–21.5)	< 0.001
Uric acid (mg/dL)	5.5 (4.5–6.7)	5.1 (4.2–6.4)	5.5 (4.6–6.8)	6.3 (5.3–7.3)	< 0.001
Total cholesterol (mg/dL)	192.0 (168–225)	190 (165–215)	192 (168–225)	217 (175–248)	< 0.001
**Oxford classification**					
M0, M1, n (%)	441, 430 (49)	220, 40 (15)	213, 312 (59)	8, 78 (91)	< 0.001
E0, E1, n (%)	479, 391 (45)	252, 8 (3.1)	220, 304 (58)	7, 79 (92)	< 0.001
S0, S1, n (%)	243, 628 (72)	160, 100 (38)	75, 450 (86)	8, 78 (91)	< 0.001
T0, T1, T2, n (%)	631, 189 (22), 51 (6)	250, 10 (5.3), 0 (0)	367, 131 (25), 27 (5.1)	14, 48 (56), 24 (28)	< 0.001
C0, C1, C2, n (%)	454, 370 (42), 46 (5)	255, 5 (1.9), 0 (0)	199, 311 (59), 14 (2.7)	0, 54 (63), 32 (37)	< 0.001
Total score	2.5 ± 1.6	0.63 ± 0.48	3.0 ± 0.78	5.2 ± 0.47	< 0.001
**Treatments**					
Corticosteroids/immunosuppressors	426 (49)	77 (30)	285 (54)	64 (74)	< 0.001
RAS inhibitors	293 (34)	65 (25)	186 (35)	42 (49)	< 0.001
**At the final follow-up**					
Observation period (years)	8 (4–10)	7.5 (4–10)	9 (4–10)	8.3 (4.5–10)	0.23
Number of patients with ESRD, n (%)	115 (13)	8 (3.1)	49 (9.3)	18 (21)	< 0.001

*O-grade* new grading system utilising the total score of each variable in the Oxford classification (MEST-C), *BMI* body mass index, *MAP* mean arterial pressure, *HPF* high-power field, *M1* mesangial hypercellularity, *E1* endocapillary hypercellularity, *S1* segmental glomerulosclerosis/adhesion, *T1* tubular atrophy/interstitial fibrosis 26–50%, *T2* 50%, *C1* cellular/fibrocellular crescents 1–25%, *C2* > 25%; RAS, renin-angiotensin system.

### Pathological classification and grades

We summed the scores for each variable in the Oxford classification for each patient, and analysed the total score of the Oxford classification for the renal prognosis with the Kaplan–Meier method and the univariate and multivariate analysis of the Cox proportional hazard regression model (Supplementary Table S1, Fig. S1 in the supplementary information). We found that the total score tended to correlate with renal prognosis. We then assigned the eight scores of 0–7 points to one of the three groups, O-grades I-III (O-grade I: total points 0–1, O-grade II: total points 2–4, O-grade III: total points: 5–7). In the O-grades, the clinical findings became significantly severer with increasing O-grades. Patients with higher O-grades were significantly older than those with lower O-grades (*P* = 0.006). The proportion of patients with proteinuria ≥ 0.5 g/day and haematuria was also significantly higher in those with higher O-grades (*P* < 0.001, *P* = 0.015, respectively). Mean arterial pressure (MAP), eGFR, uric acid, and total cholesterol worsened significantly with increasing O-grades (*P* < 0.001) (Table 1b). These results indicated that our determined grading was reasonable.

### Renal survival

**Figure 1:**
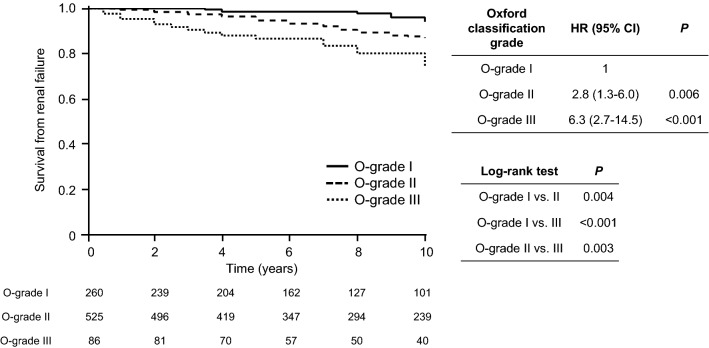
Kaplan–Meier renal survival curves of patients with IgA nephropathy according to the O-grade. The renal survival rates were 94.1%, 86.9%, and 74.1% for O-grades I, II, and III, respectively (P < 0.001). *O-grade* new grading system utilising the total score of each variable in the Oxford classification (MEST-C).

Figure 1 shows the renal survival rate according to the O-grades. The 10-year renal survival rate by the Kaplan–Meier method was 94.1%, 86.9%, and 74.1% for O-grades I, II, and III, respectively. The renal survival rates decreased as the O-grade increased (log-rank test: *P* < 0.001). In the Cox proportional hazards regression model, the HRs for O-grades II and III with O-grade I as the reference were 2.8 (95% CI 1.3–6.0; *P* = 0.006) and 6.3 (95% CI 2.7–14.5; *P* < 0.001), respectively.

Age, sex, body mass index (BMI), MAP, eGFR, proteinuria, O-grade, and corticosteroids/immunosuppressors were significantly associated with ESRD in the univariate analysis (Table 2). In the multivariate analysis including these significant independent factors related to renal prognosis, three clinical variables, including MAP (HR 1.31; 95% CI 1.07–1.62; *P* < 0.001, every 10-mm Hg increase), eGFR (HR 4.88; 95% CI 3.26–7.33; *P* < 0.001, every 30-ml/min/1.73 m^2^ decrease), and proteinuria (HR 1.17; 95% CI 1.12–1.21; *P* < 0.001, every 0.5-g/day increase), O-grade (HR 1.63; 95% CI 1.11–2.38; *P* = 0.012, every O-grade increase), and corticosteroids/immunosuppressors (HR 0.46; 95% CI 0.27–0.80; *P* = 0.005) were independent factors contributing to the renal prognosis.

**Table 2:** Correlations between the clinical and pathological features and ESRD in the univariate and multivariate Cox regression analyses.

Baseline data	Univariate hazard ratio (95% CI)	*P *value	Multivariate hazard ratio (95% CI)	*P *value
**Clinical findings**				
Age (baseline < 20 years, per 10-year increase)	1.26 (1.06–1.50)	0.01	0.82 (0.66–1.03)	0.09
Sex (male, vs. female)	2.08 (1.32–3.29)	0.002	1.38 (0.84–2.27)	0.20
BMI (baseline < 20 kg/m^2^, per 1-kg/m^2^ increase)	1.09 (1.01–1.17)	0.03	0.95 (0.88–1.03)	0.25
MAP (baseline < 90 mmHg, per 10-mmHg increase)	1.28 (1.04–1.58)	< 0.001	1.31 (1.07–1.62)	0.009
**Laboratory findings**				
eGFR (baseline ≥ 90 mL/min, per 30-ml/min decrease)	4.33 (3.13–6.05)	< 0.001	4.88 (3.26–7.33)	< 0.001
Proteinuria (baseline ≤ 0.5 g/day, per 0.5-g/day increase)	1.13 (1.10–1.16)	< 0.001	1.17 (1.12–1.21)	< 0.001
Haematuria (baseline < 5/HPF, per 25/HPF increase)	0.98 (0.85–1.12)	0.80		
**Histological findings**				
Oxford classification grade (O-grade)	2.43 (1.66–3.56)	< 0.001	1.63 (1.11–2.38)	0.012
**Treatments**				
Corticosteroids/immunosuppressors	0.67 (0.42–1.06)	0.08	0.46 (0.27–0.80)	0.005
RAS inhibitors	1.01 (0.63–1.62)	0.96	0.69 (0.40–1.18)	0.17

*95% CI* 95% confidence interval, *BMI* body mass index, *MAP* mean arterial blood pressure, *HPF* high-power field, *ESRD* end-stage renal disease, *O-grade* a new grading system utilising the total score of each variable in the Oxford classification (MEST-C) and the renal survival rate, *RAS* renin-angiotensin system.

### Combination of pathological grades and clinical classification

The patients were further classified into nine groups according to the three O-grades combined with the three C-grades, and their renal prognosis was compared (Table 3). In a group with O-grade I and C-grade I, the incidence of ESRD was 0%, and in a group with O-grade I and C-grade II, the incidence of ESRD was 2.6%, this group was assigned as the reference group to compare increasing O-grades. In the univariate Cox regression analysis, the HRs of each group compared to the reference group showed an increasing tendency, especially those of the groups with higher O-grades and C-grades. In a group with O-grade III and C-grade III, the incidence of ESRD was 33%, and the HR was 15.2 (95% CI 3.5–67; *P* < 0.001); this group had the severest renal prognosis when compared to the reference group.

**Table 3:** Classification and the risk of progression to ESRD according to the combination of the clinical-grade and O-grade.

Clinical grade	Oxford classification grade		
	O-grade I	O-grade II	O-grade III
**C-grade I**			
Incidence of ESRD (%)	0/153 (0)	3/163 (1.8)	1/7 (14)
HR (95% CI)		0.79 (0.13–4.7)	6.3 (0.57–69)
*P *value		0.80	0.13
**C-grade II**			
Incidence of ESRD (%)	2/78 (2.6)	12/250 (4.8)	3/37 (8.1)
HR (95% CI)	1	1.9 (0.43–8.6)	3.0 (0.50–18)
*P *value		0.39	0.23
**C-grade III**			
Incidence of ESRD (%)	6/19 (32)	32/105 (30)	14/42 (33)
HR (95% CI)	13.3 (2.7–66)	13.7 (3.3–57)	15.2 (3.5–67)
*P *value	0.0015	< 0.001	< 0.001

*O-grade* a new grading system utilising the total score of each variable in the Oxford classification (MEST-C) and the renal survival rate, *C-grade* a grading system which was based on the Japanese clinical classification, *HR* hazard ratio, *95% CI* 95% confidence interval, *ESRD* end-stage renal disease.

## Discussion

In this study, we classified 871 IgAN patients into three groups according to the total MEST-C score in the Oxford classification (O-grade); this O-grade classification system was significantly associated with the renal prognosis. Moreover, the combination of the O-grade with the clinical classification of eGFR and proteinuria correlated with the renal prognosis. To the best of our knowledge, this is the first study that described the relevance of the total MEST-C score on renal prognosis. Using our interpretation of the Oxford classification, predicting the renal prognosis became straightforward, especially when combined with the clinical findings.

In the Oxford classification, various pathological lesions of IgAN and their reproducibility were analysed, and the split system was adopted. Its usefulness has been widely recognised after the analyses conducted by many validation studies^11–16^. However, in the Oxford classification, although it allows a more straightforward evaluation of each glomerular lesion, the evaluation of the total severity of the pathological findings was difficult^5,7,8^. On the other hand, in the Japanese histological classification, the total severity of pathological findings can be easily evaluated; however, the evaluation of each lesion was challenging due to its lumped grading system^6^. Therefore, in this study, we utilised the advantages of both the Oxford and Japanese histological classifications.

**Figure 2:**
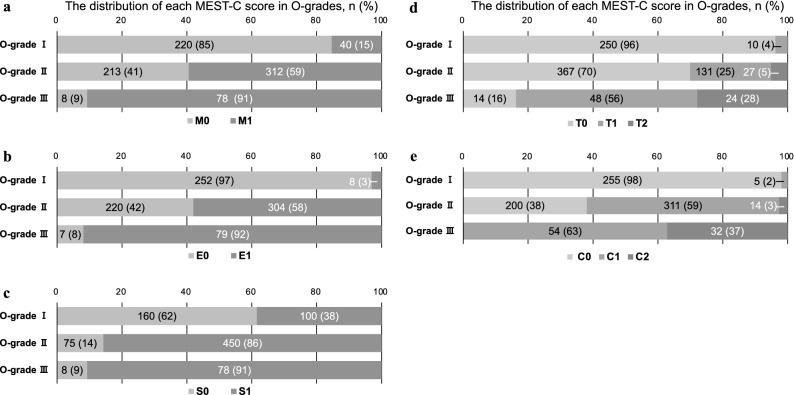
Distribution of MEST-C score for patients with IgA nephropathy in O-grades. (**a**–**e**) The number and the ratio of patients with the renal biopsy-lesions of M, E, S, T, and C in O-grade I–III, respectively. *O-grade* new grading system utilising the total score of each variable in the Oxford classification (MEST-C), *M1* mesangial hypercellularity, *E1* endocapillary hypercellularity, *S1* segmental glomerulosclerosis/adhesion, *T1* tubular atrophy/interstitial fibrosis 26–50%, *T2* 50%, *C1* cellular/fibrocellular crescents 1–25%, *C2* 25%.

One of the most noteworthy findings in our study is that the increase of the O-grades, classified according to the total score of the Oxford classification, was associated with worsening of the renal prognosis in the Kaplan–Meier analysis. Moreover, the univariate Cox proportional hazard regression model showed that the HR of O-grades II and III against O-grade I as the reference were 2.8 (95% CI 1.3–6.0) and 6.3 (95% CI 2.7–14.5), respectively. A similar tendency was observed in the odds ratio of the Japanese histological classification^6^. Previous reports indicated that each lesion, with M^12,15,16^, E^11,13^, S^11,13,16^, T^11–16^, and C^14^ in the Oxford classification, was significantly associated with the renal prognosis. However, these results were variable because each cohort differed in terms of race, eligibility criteria, treatments, and endpoints. In our previous report, the Oxford classification was useful to predict the renal prognosis, similar to that reported by other previous studies of IgAN patients without corticosteroid treatment, and the E, S, T, and C scores predicted the renal prognosis. However, the prognosis of patients with E1, S1, and C1 scores improved with corticosteroid treatment, and the multivariate analysis indicated that only T score and several clinical factors, such as proteinuria and eGFR, were the independent predictors of renal prognosis, but M, E, S, and C scores were not^17^. According to the findings of this report, the Oxford classification was useful in predicting the renal prognosis. However, each MEST-C score is affected by several confounders, and the total MEST-C score might comprehensively show the degree of the disease severity, which may unify the differences in the results from each cohort. Moreover, some previous studies evaluated the pathological lesions with the “split” system, and the total scores consisted of the sum of the scores of each lesion to evaluate the disease severity^18–21^. The results of these previous reports support our grading system of classifying the total score of 0–7 points of the Oxford classification into three grades of 0–1, 2–4, and 5–7 points according to the incidence of ESRD. In addition, the number of patients with each M1, E1, S1, T1, T2, C1, and C2 increased, as the O-grade increased from I to III, and we found that M, E, S, and C scores accounted for as much as T-score in O-grades II and III (Fig. 2). Therefore, we judged that, in addition to T-score, all other MEST-C scores were also involved in O-grade prediction of renal survival.

Regarding the clinical classification, several reports indicated that eGFR^2,4,22–26^, proteinuria^2,4,22,24–29^, hypertension^2,23–28^, and age^2,4,24–26^ at the time of renal biopsy were associated with ESRD. In this study, we identified three clinical findings, i.e., MAP, eGFR, and proteinuria, as risk factors for ESRD in the multivariate analysis, and the O-grade, a histological finding, was also found as a risk factor. The appropriate treatment for IgAN was generally decided depending on the clinical and histological findings, and the renal prognosis was also predicted based on these data. The clinical and histological classifications established by these findings facilitate more accurate decision-making by nephrologists. Therefore, both histological and clinical findings are essential for the management of IgAN. Although a new international risk-prediction tool with both histological and clinical parameters has been published^30^ and widely evaluated, in this study, O-grade was analysed in a multivariate analysis using the clinical factors and treatments adopted in the international risk-prediction tool. The combination with clinical-grade (C-grade) classified with eGFR and proteinuria as clinical factors could predict renal prognosis by considering the clinical and pathological factors simultaneously, as well as the international risk prediction tool. Moreover, the present study adopted a harder endpoint of introducing renal replacement therapies for a more extended period of 10 years.

Taking the abovementioned findings into account, we analysed the renal prognosis using the combination of O-grade and C-grade (Japanese histological classification) classification systems. Among the renal prognoses of the nine groups classified according to the combination of the O-grades and C-grades, the highest HR was observed in the group with O-grade III and C-grade III, which showed the severest form of the disease. Moreover, HR also tended to increase with increasing O-grades and C-grades. These results suggested that the combination of the O-grades and C-grades could predict the progression to ESRD more accurately than either grade alone. Some studies have reported that the combination of the classifications of clinical and pathological findings has predicted the prognosis of IgAN^24,25^. Similarly, it is suggested that our method also predicts renal prognosis well.

This study has several limitations. First, this study was conducted retrospectively at a single institution in Japan. Even in the past validation studies, differences in race, eligibility criteria, clinical background, and the frequency of each lesion in the Oxford classification were present^11–16^. If the validation studies were performed more accurately, the studies should be done with the same or with some established criteria. Second, various treatments were not considered in this study because of the long-term observation period, although the treatment was also an essential factor for IgAN, and the classifications should be used for the appropriate selection of each treatment. Thus, it is necessary to evaluate in future studies whether the O-grade system is useful for determining the therapeutic indications.

In conclusion, our data suggest that the O-grade, which is classified into three grades according to the total score of the Oxford classification, was associated with renal prognosis. Moreover, the O-grade could be useful for predicting renal prognosis more accurately when combined with the Japanese clinical classification that utilised eGFR and proteinuria. The O-grade could facilitate a straightforward evaluation of the total activity and chronicity of IgAN and the prediction of renal prognosis in patients with IgAN.

## Methods

### Study design and participants

Patients were identified from a database of all renal biopsies performed at the Tokyo Women’s Medical University, Japan. All patients aged > 16 years who were diagnosed with IgAN by renal biopsy between 1974 and 2015 were eligible for this retrospective cohort study. Patients with systemic diseases, such as IgA vasculitis, diabetes, systemic lupus erythematosus, or liver cirrhosis, those with missing data, and those with < 8 glomeruli collected during the renal biopsy were excluded. Data of 1147 patients were collected, and after applying the inclusion and exclusion criteria, 276 patients were excluded. The remaining 871 patients were followed for up to 10 years. The baseline and follow-up clinical data, such as sex, age, duration from onset to biopsy, BMI, MAP, proteinuria, haematuria, serum creatinine level, eGFR, blood urea nitrogen (BUN) level, uric acid level, total cholesterol level, observation period, and the number of patients with end-stage renal disease (ESRD), were obtained from the patients’ medical records according to a unified protocol. MAP was identified as the diastolic pressure plus one-third of the pulse pressure. Proteinuria was defined as the amount of protein in a 24-h urine sample collected at the time of renal biopsy, and haematuria was defined as the presence of urinary red blood cells in the high-power field (HPF) on microscopic examination of urinary sediments. The eGFR was calculated based on the Japanese modified isotope dilution mass spectrometry-traceable 4-variable Modification of Diet in Renal Disease Study equation^31^. The observation period was defined as the duration between the renal biopsy and the last follow-up, death, or the onset ESRD. The primary outcome was ESRD requiring renal replacement therapies (permanent haemodialysis, peritoneal dialysis, or renal transplantation).

This study was conducted following the guidelines stipulated in the Declaration of Helsinki and was approved by the Medical Ethics Committee at the Tokyo Women’s Medical University (# 5104-R). We have obtained written informed consent for renal biopsy from all patients, and clinical data were obtained from all recently recruited patients, who had the opportunity to opt-out from this study.

### Pathology

IgAN was diagnosed by a renal biopsy demonstrating the predominant mesangial deposition of IgA. The lesions were recorded according to the Oxford classification: mesangial cellularity score, ≤ 0.5 (M0) and > 0.5 (M1); the presence of endocapillary proliferation, absent (E0) and present (E1); segmental glomerulosclerosis/adhesion, absent (S0) and present (S1); the severity of tubular atrophy/interstitial fibrosis, ≤ 25% (T0), 26–50% (T1), and > 50% (T2); and cellular/fibrocellular crescents, absent (C0), 1–25% (C1), and > 25% (C2)^5,8,9^.

All patients were first assigned a total score of 0–7, which was the sum of each Oxford classification (MEST-C) score, and then, they were simply classified into one of three groups (O-grades I, II, and III): O-grade I, a total score of 0–1; O-grade II, a total score of 2–4; and O-grade III, a total score of 5–7.

### Clinical classification

All patients were classified into the following grades according to the clinical data available at the time of renal biopsy, which were based on the Japanese clinical classification (C-grade): C-grade I, proteinuria < 0.5 g/day; C-grade II, proteinuria ≥ 0.5 g/day and eGFR ≥ 60 ml/min/1.73 m^2^; and C-grade III, proteinuria ≥ 0.5 g/day and eGFR < 60 ml/min/1.73 m^2^^10^.

### Statistical analyses

The Anderson–Darling test was used to evaluate the normal distribution of the quantitative variables. The results were expressed as means ± standard deviations if normally distributed; otherwise, they were reported as medians with interquartile ranges for non-normal distributions. The baseline characteristics of the three pathological groups (O-grades I, II, and III) were compared using the analysis of variance as a parametric test or the Kruskal–Wallis test as a nonparametric test. Comparisons between proportions were made using the chi-square tests. The three groups were analysed by using the Kaplan–Meier method and compared with the univariate Cox proportional hazards regression model and the log-rank test for the renal prognosis. The baseline characteristics of the three groups were subjected to the univariate and multivariate Cox regression analyses to assess the impact of the factors on the renal prognosis. The univariate Cox regression analysis was also performed for the nine groups, which was the combination of the three pathological and three clinical classification groups, and the hazard ratio (HR) was calculated with the group with C-grade I and O-grade I used as the reference category^10^. The HRs were expressed with 95% confidence intervals (95% CI). *P* values < 0.05 were considered statistically significant. The analyses were performed with JMP version 15 (SAS Institute Inc. Cary, NC, USA).

## Supplementary Information

Supplementary Information

## Data Availability

The clinical data, the study protocol, and statistical analysis plan that support this research are available from the corresponding author, T.M., upon reasonable request, and with restrictions on information that could compromise the privacy of the patients.
