# Supplementary material for: Grading system utilising the total score of Oxford classification for predicting renal prognosis in IgA nephropathy
Source: Sci Rep. 2021 Feb 11;11:3584. doi: 10.1038/s41598-021-82967-x (PMC7878747; doi:10.1038/s41598-021-82967-x)
Supplement: Supplementary file 1 — Supplementary Information [file 41598_2021_82967_MOESM1_ESM.pdf]

Supplementary information

Grading system utilising the total score of Oxford classification for predicting renal prognosis in IgA nephropathy

Yoei Miyabe[1], Kazunori Karasawa[1], Kenichi Akiyama[1], Shota Ogura[1], Tomo Takabe[1], Naoko Sugiura[1], Momoko Seki[1], Yuko Iwabuchi[1], Norio Hanafusa[2], Keiko Uchida[1], Kosaku Nitta[1], Takahito Moriyama[1*].

[1]Department of Nephrology, Tokyo Women's Medical University, 8-1 Kawada-Cho, Shinjuku-ku, Tokyo, 1628666, Japan

Supplementary Figure S1. Kaplan-Meier renal survival curves for patients with IgA nephropathy in the total score of Oxford classification. The renal survival rates by the Kaplan-Meier method were 97.1%, 92.1%, 91.2%, 86.6%, 83.8%, 74.0%, 74.3%, and 100% for 0 to 7 points in the Oxford classification total score, respectively.

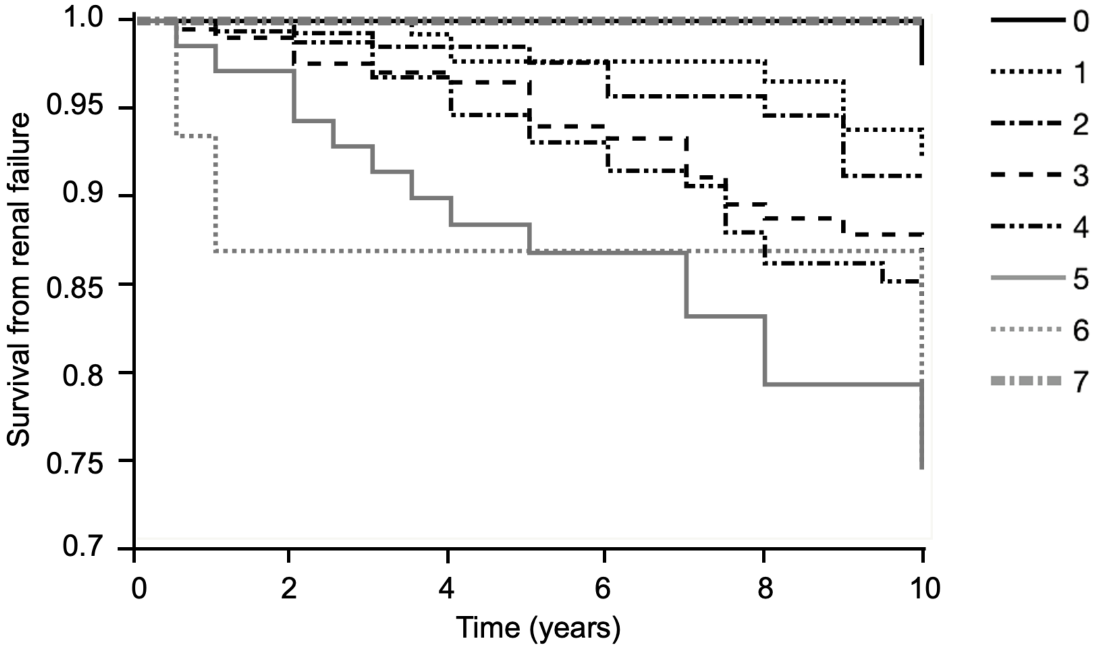

0 points	97	87	78	60	45	39
1 point	163	152	126	102	84	62
2 points	152	142	115	100	86	74
3 points	206	198	171	134	110	86
4 points	167	156	133	113	98	79
5 points	69	67	58	49	42	32
6 points	15	12	10	8	8	7
7 points	2	2	2	2	2	1

Supplementary Table S1. Correlations between clinical and pathological features and ESRD in the univariate and multivariate Cox regression analyses with total score of the Oxford classification

Baseline data	Univariate hazard ratio (95% CI)	*P-value*	Multivariate hazard ratio (95% CI)	*P-value*
Clinical findings				
Age (baseline <20 years, per 10-year increase)	1.26 (1.06-1.50)	0.01	0.80 (0.64-1.01)	0.06
Sex (male, vs female)	2.08 (1.32-3.29)	0.002	1.42 (0.86-2.32)	0.17
BMI (baseline <20 kg/m^2, per 1-kg/m^2 increase)	1.09 (1.01-1.17)	0.03	0.95 (0.87-1.03)	0.20
MAP (baseline <90 mmHg, per 10-mmHg increase)	1.28 (1.04-1.58)	<0.001	1.32 (1.07-1.62)	0.009
Laboratory findings				
eGFR (baseline ≥90 ml/min, per 30-ml/min decrease)	4.33 (3.13-6.05)	<0.001	4.96 (3.29-7.48)	<0.001
Proteinuria (baseline <0.5 g/day, per 0.5-g/day increase)	1.13 (1.10-1.16)	<0.001	1.17 (1.12-1.21)	<0.001
Haematuria (baseline <5/HPF, per 25/HPF increase)	0.98 (0.85-1.12)	0.80		
Histological findings				
Total score of the Oxford classification	1.46 (1.25-1.71)	<0.001	1.33 (1.13-1.56)	<0.001
Treatments				
Corticosteroids/immunosuppressors	0.67 (0.42-1.06)	0.08	0.43 (0.25-0.74)	0.002
RAS inhibitors	1.01 (0.63-1.62)	0.96	0.66 (0.39-1.13)	0.13

95% CI, 95% confidence interval; BMI, body mass index; MAP, mean arterial blood pressure; HPF, high-power field; O-grade, a new grading system utilizing the total score of each variable in the Oxford classification (MEST-C) and the survival rate; RAS, renin-angiotensin system.
